# Identification of novel pathways involved in the pathogenesis of human adamantinomatous craniopharyngioma

**DOI:** 10.1007/s00401-012-0957-9

**Published:** 2012-02-18

**Authors:** Cynthia L. Andoniadou, Carles Gaston-Massuet, Rukmini Reddy, Ralph P. Schneider, Maria A. Blasco, Paul Le Tissier, Thomas S. Jacques, Larysa H. Pevny, Mehul T. Dattani, Juan Pedro Martinez-Barbera

**Affiliations:** 1 Neural Development Unit, UCL Institute of Child Health, 30 Guilford Street, London, WC1N 1EH UK; 2 Telomeres and Telomerase Group, Molecular Oncology Program, Spanish National Cancer Research Centre, 28029 Madrid, Spain; 3 Department of Histopathology, Great Ormond Street Hospital for Children, London, WC1N 3JH UK; 4 Department of Cell and Developmental Biology, Neuroscience Center, University of North Carolina, Chapel Hill, NC USA; 5 Developmental Endocrinology Research Group, UCL Institute of Child Health, London, WC1N 1EH UK

**Keywords:** Adamantinomatous, Craniopharyngioma, β-Catenin, Mouse, Pituitary tumour, Stem cells

## Abstract

Activating mutations in the gene encoding β-catenin have been identified in the paediatric form of human craniopharyngioma (adamantinomatous craniopharyngioma, ACP), a histologically benign but aggressive pituitary tumour accounting for up to 10% of paediatric intracranial tumours. Recently, we generated an ACP mouse model and revealed that, as in human ACP, nucleocytoplasmic accumulation of β-catenin (β-cat^nc^) and over-activation of the Wnt/β-catenin pathway occurs only in a very small proportion of cells, which form clusters. Here, combining mouse genetics, fluorescence labelling and flow-sorting techniques, we have isolated these cells from tumorigenic mouse pituitaries and shown that the β-cat^nc^ cells are enriched for colony-forming cells when cultured in stem cell-promoting media, and have longer telomeres, indicating shared properties with normal pituitary progenitors/stem cells (PSCs). Global gene profiling analysis has revealed that these β-cat^nc^ cells express high levels of secreted mitogenic signals, such as members of the SHH, BMP and FGF family, in addition to several chemokines and their receptors, suggesting an important autocrine/paracrine role of these cells in the pathogenesis of ACP and a reciprocal communication with their environment. Finally, we highlight the clinical relevance of these findings by showing that these pathways are also up-regulated in the β-cat^nc^ cell clusters identified in human ACP. As well as providing further support to the concept that pituitary stem cells may play an important role in the oncogenesis of human ACP, our data reveal novel disease biomarkers and potential pharmacological targets for the treatment of these devastating childhood tumours.

**Electronic supplementary material:**

The online version of this article (doi:10.1007/s00401-012-0957-9) contains supplementary material, which is available to authorized users.

## Introduction

Adamantinomatous craniopharyngioma (ACP) is the most common non-neuro-epithelial brain tumour in children [31, 43, 47]. Although not metastatic and histologically benign, ACP is invasive and prone to recurrence after surgery, the conventional mode of treatment. Adamantinomatous craniopharyngioma often behaves aggressively with invasion of the hypothalamus and visual pathways. Therefore, total resection of the tumour without damage to vital surrounding structures such as the hypothalamus and optic chiasm is not always possible. In these children, radical excision is associated with unacceptable morbidity and mortality whilst subtotal resection without adjuvant radiotherapy predisposes to a high (>60%) 3-year recurrence risk and further hypothalamic and visual compromise [34, 38, 44]. Although a conservative surgical approach with adjuvant radiotherapy for residual tumour has been recently adopted, both tumour recurrence and treatment-associated morbidity are still high. Consequences of the tumour and its treatment include obesity with associated Type 2 diabetes mellitus, learning difficulties, visual impairment and panhypopituitarism, which can be life-threatening. This poses a heavy burden to parents and carers as well as a heavy cost for health services.

A crucial role for Wnt/β-catenin signalling in the aetiology of ACP has been firmly established. Activating mutations in the gene encoding β-catenin (*CTNNB1*) have been identified in the majority of samples of human ACP [9, 51]. Recently, phenotypic analysis of a mouse model (*Hesx1* ^*Cre/*+^;*Ctnnb1* ^*lox(ex3)/*+^) expressing a mutant form of β-catenin that cannot be degraded, leading to over-activation of the pathway, has confirmed that these mutations, rather than a second hit, are causative of the tumours [18]. A characteristic histological finding in both human and mouse ACP is the restricted nucleocytoplasmic accumulation of β-catenin and over-activation of the Wnt/β-catenin pathway in very few cells that form clusters (β-cat^nc^ clusters). Despite harbouring the tumorigenic mutation in the β-catenin gene, all other cells only show the normal β-catenin staining in the cytoplasmic membrane without any nucleocytoplasmic accumulation (non-cluster β-cat^m^ cellular component of ACP) [18, 25, 26, 32]. Beyond the relevant diagnostic value of this unique histological feature to distinguish ACP from other pituitary tumours, little is known about the reason for this specificity or the relevance of the cluster cells in the disease. Recent evidence points towards a role in tumour progression and invasiveness into the brain [8, 27–29]. A deeper analysis of these cellular structures may provide novel insights into the pathogenesis of ACP resulting in the identification of new disease biomarkers and pharmacological targets.

An open question is the contribution of pituitary progenitors/stem cells (PSCs) in the aetiology and pathogenesis of ACP. Previously, we have demonstrated that one of the initial effects of mutated β-catenin is the increase of PSCs in the ACP murine model compared with control pituitaries. At late gestation and early postnatal stages, a proportion of β-catenin-accumulating cells within the clusters express the stemness marker SOX2 in the murine pre-tumoral pituitary [14, 18]. However, SOX2 is not expressed in the β-catenin accumulating clusters in human ACP, but rather in sporadic cells within the tumour [17]. This raises the question of what the connection is between the human and mouse β-catenin-accumulating clusters.

In this study, we have used the ACP mouse model to investigate the pathogenesis and the possible involvement of PSCs in the aetiology of human ACP. We demonstrate that β-catenin-accumulating cluster cells have functional and molecular characteristics of pituitary progenitors/stem cells. We present their global gene expression profile and reveal novel genes and signalling pathways expressed in both mouse and human ACP. This molecular analysis highlights interplay between clusters and surrounding cells through the secretion of signals involved in proliferation, survival, stem cell maintenance, cell migration and tumorigenesis.

## Materials and methods

### Mice

The *Ctnnb1* ^*lox(ex3)/*+^, *Hesx1*-*Cre* and *BAT*-*gal* mice have been previously described [2, 24, 41].

### X-gal staining, in situ hybridisation and immunostaining

Wholemount X-gal staining, immunostaining and in situ hybridisation on 8 μm paraffin sections were performed as previously described [2, 18]. Samples of human ACP were obtained from the Department of Histopathology at Great Ormond Street Hospital for Children. At least three pituitaries or tumours and between two and six slides were analysed. As negative controls, sections were hybridized with sense riboprobes for in situ hybridisation or secondary antibody alone for immunohistochemistry/immunofluorescence.

### Immuno-FISH

Paraffin tissue slides were deparaffinised and underwent citrate antigen retrieval, before a fluorescent is situ hybridisation was performed as previously described [49], After washing, the slides were incubated overnight with an anti β-catenin antibody (Sigma, dilution 1:100). The primary antibody was detected with an Alexa 488-conjugated secondary antibody before DNA counterstaining with DAPI in a 4 μg/ml solution in PBS. The slides were mounted using Vectashield (Vector Laboratories).

The β-catenin/telomere immuno-FISH 8-bit pictures were acquired with a Leica TCS-SP5 (AOBS) high-resolution confocal microscope. A 63×-Leica immersion objective was used with an additional magnification of 2.5×. Stacks of seven pictures were taken with a step size of 0.8 μm. Those were further maximum projected by the LAS AF software (Leica) for analysis. Fluorescence analysis of intensities was performed with Definiens Developer XD1.8 software (Definiens). The β-cat^nc^ cluster and the β-cat^m^ surrounding cells used for the telomere analysis were selected by hand according to the β-catenin immunofluorescence staining.

### Flow sorting

The β-catenin-accumulating cellular fraction (β-cat^nc^ cluster cells) was purified from surrounding (β-cat^m^) cells of *Hesx1* ^*Cre/*+^;*Ctnnb1* ^*lox(ex3)/*+^;*BAT*-*gal* pituitaries by flow sorting taking advantage of their activation of the *BAT*-*gal* reporter. The *BAT*-*gal* transgene expresses *lacZ* (encoding β-galactosidase) under the regulation of TCF/LEF binding sites in cells with activated Wnt/β-catenin signalling [41]. For the assessment of the colony-forming potential of β-cat^nc^ cluster cells, three independent flow-sorting experiments were performed using a total of 17 *Hesx1* ^*Cre/*+^;*Ctnnb1* ^*lox(ex3)/*+^;*BAT*-*gal* pituitaries at 18.5 dpc. In brief, cells were dissociated by incubation for 4 h at 37°C in an enzyme mix containing collagenase type II (Sigma), trypsin (Gibco) and DNase I (Worthington) in HBSS (Gibco). After washing in HBSS, the cells were manually dissociated and then treated using the CMFDG kit (Invitrogen) according to manufacturer’s recommendations, to yield a fluorescent product when the CMFDG substrate is cleaved by β-galactosidase. The cells were flow-sorted immediately in PBS containing 1% fetal calf serum and 25 mM HEPES using a MoFlo XDP (Beckman Coulter, Fullerton, California, USA). GFP fluorescence was detected using a 530/40 filter, and dead and auto-fluorescent cells were excluded using propidium iodide (Invitrogen) using a 613/20 filter. The analysis for cell sorting was carried out on Summit software (Dako). Flow sorting of cells from *Sox2* ^*eGFP/*+^ pituitaries was carried out as above, without CMFDG treatment or addition of propidium iodide. The data presented were obtained from three independent experiments using a total of 19 *Sox2* ^*eGFP/*+^ mice of 6–8 weeks of age. For subsequent culture, the cells were collected in culture medium and for RNA isolation, in RLT lysis buffer (Qiagen), flash frozen and stored at −80°C until processed.

### Cell culture

Single cells were cultured as adherent colonies using methods described by Gleiberman et al. [20] and plated at a clonal density of 20 cells per well of a 96-well plate (roughly 60 cells per cm^2^), or graded densities (1,000–4,000 cells per well) in six-well plates. The colonies were fixed after 1 week and stained with haematoxylin as previously described [18].

### RNA extraction, microarray methods and quantitative real-time PCR

Total RNA isolation from pituitaries and flow-sorted cells was performed as described using the RNeasy Micro Kit (Qiagen) [3]. Approximately 3,000 β-cat^nc^ cluster cells were obtained from seven independent flow-sorting experiments of dissociated and CMFDG treated *Hesx1* ^*Cre/*+^;*Ctnnb1* ^*lox(ex3)/*+^;*BAT*-*gal* pituitaries (*n* = 15 mice). Linear amplification of purified RNA was carried out using the Ovation Pico WTA System (NuGEN), following manufacturer’s recommendations, yielding approximately 5.5 μg of polyadenylated RNA. From this, 3.0 μg were used for microarray analysis, using standard Affymetrix labelling and hybridization protocols for hybridization on GeneChip Mouse 430_2 arrays. The remaining 2.5 μg were reverse transcribed to cRNA with Omniscript RT (Qiagen) and random hexamers (Promega) and used for qRT-PCR. RNA from *Sox2* ^*eGFP/*+^ flow-sorted pituitaries was isolated and processed as described and used only for qRT-PCR analyses. Reactions were run in triplicate on an ABI 7500 Fast cycler using MESA Blue reagent (Eurogentec) and repeated for a minimum of four independent samples for each cell type. Primer sequences are available on request. Results were analysed using the ΔΔCt method.

## Results

### β-Catenin-accumulating cells from tumorigenic murine pituitaries share properties with normal pituitary progenitors/stem cells

We have previously shown that tumorigenic pituitaries from *Hesx1* ^*Cre/*+^;*Ctnnb1* ^*lox(ex3)/*+^ mice contain higher numbers of cells with clonogenic potential, this being the ability to form colonies comprised of undifferentiated progenitors when cultured in stem-cell-promoting media [18]. However, it is not known whether these clonogenic cells correspond to the population accumulating β-catenin in the nucleus and cytoplasm (β-cat^nc^ clusters) or those showing only the normal membranous or sub-membranous β-catenin localization (β-cat^m^) without any nucleocytoplasmic accumulation. This is clinically relevant since these two cell populations exist in human ACP and indeed, the presence of β-cat^nc^ clusters is used as a diagnostic histopathological feature that differentiates human ACP from other brain tumours of the sellar and suprasellar areas of the brain [26]. To provide insights into the cellular nature of β-cat^nc^ clusters, we carried out detailed molecular and cell culture analyses in the mouse model.

**Fig. 1:**
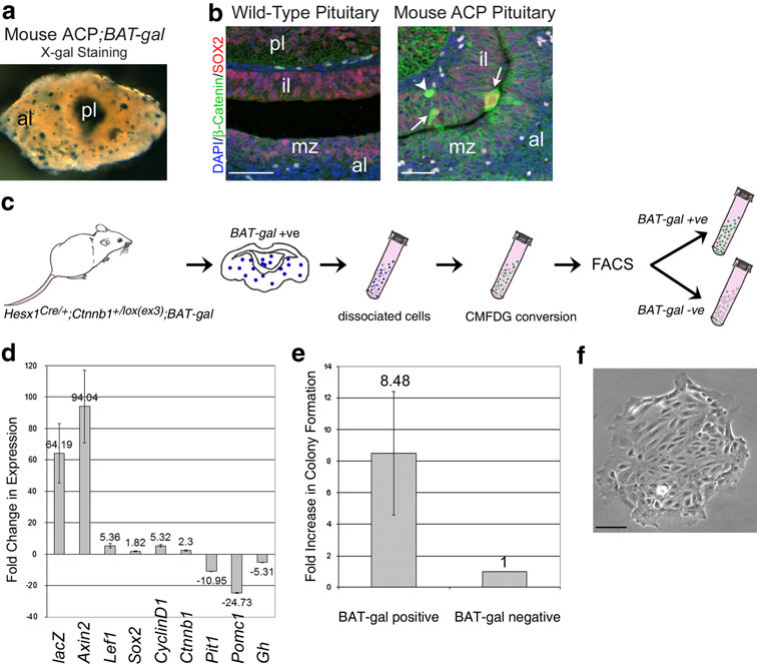
Purification of β-catenin-accumulating cells from *Hesx1* ^*Cre/*+^;*Ctnnb1* ^*lox(ex3)/*+^;*BAT*-*gal* pituitaries. **a** X-gal staining of a *Hesx1* ^*Cre/*+^;*Ctnnb1* ^*lox(ex3)/*+^;*BAT*-*gal* mutant pituitary showing the activation of the *BAT*-*gal* reporter and expression of β-galactosidase in clusters within the anterior lobe. The posterior lobe is highly stained and was surgically removed prior to the purification procedure. **b** Double immunofluorescence staining against β-catenin (*green*) and SOX2 (*red*) in wild-type and *Hesx1* ^*Cre/*+^;*Ctnnb1* ^*lox(ex3)/*+^ (mouse ACP) pituitaries. Note the nucleocytoplasmic accumulation of β-catenin in very few cells that form clusters in the *Hesx1* ^*Cre/*+^;*Ctnnb1* ^*lox(ex3)/*+^ pituitary (*arrows*), whilst the majority of the cells show normal cytoplasmic staining as in the control pituitary. SOX2 is expressed in cells of the marginal zone around the lumen in the control but in *Hesx1* ^*Cre/*+^;*Ctnnb1* ^*lox(ex3)/*+^ pituitaries, some cells within the clusters also express SOX2 (*arrows*). Note a cluster that is negative for SOX2 expression (*arrowhead*). **c** Scheme of the strategy to purify the β-catenin-accumulating cell clusters. Triple heterozygous pituitaries (only anterior and intermediate lobes) are dissociated into single cell suspension, treated with CMFDG (a fluorogenic substrate for β-galactosidase) and subjected to flow-sorting. The two fractions are used for qRT-PCR analysis, gene profiling and stem cell culture. **d** qRT-PCR comparing BAT-gal^+ve^ (clusters) versus BAT-gal^−ve^ (non-clusters) cell fractions confirming the efficiency of the purification. Fold-changes in expression indicated on the *y*-axis, where >0 means higher expression in BAT-gal^+ve^ and <0 higher in BAT-gal^−ve^. Cluster cells fit an undifferentiated profile (high *Sox2* and low *Pit1*, *Pomc1* and *Gh*) and show activation of the Wnt/β-catenin pathway (high *lacZ*, *Axin2*, *Lef1* and *CyclinD1*). **e**, **f** The BAT-gal^+ve^ fraction contains 8.48 times more cells with clonogenic potential (progenitors/stem cells, PSCs) (**e**) able to form single cell-derived colonies when cultured in stem cell-promoting media (**f**). *pl* posterior lobe, *il* intermediate lobe, *al* anterior lobe, *mz* marginal zone. *Scale bars* **b** 50 μm, **f** 100 μm

As activation of the Wnt/β-catenin pathway, as assessed by the expression of Wnt/β-catenin target genes, is restricted to the β-cat^nc^ clusters in both mouse and human ACP [29, 52], we used a genetic tool in mouse to enable their purification. Specifically, we employed the *BAT*-*gal* mouse strain, a reporter line of active Wnt/β-catenin signalling, which expresses *lacZ*, encoding β-galactosidase, under the control of TCF/LEF binding sites [41]. In *Hesx1* ^*Cre/*+^;*Ctnnb1* ^*lox(ex3)/*+^;*BAT*-*gal* triple heterozygous pituitaries, β-galactosidase activity is present in clusters of cells (Fig. 1a), which corresponds to the cells that accumulate nucleocytoplasmic β-catenin and, a proportion of which, also express SOX2 (Fig. 1b) [18]. This enzymatic activity was used to generate a luminous product after treatment and fluorescent cells from dissociated pituitaries were sorted by flow cytometry resulting in the purification of two cell fractions: (1) BAT-gal^+ve^ that correspond to β-cat^nc^ cluster cells and (2) BAT-gal^−ve^ containing all the remaining cells from the intermediate and anterior pituitary lobes (β-cat^m^ cells) (Fig. 1c). The efficiency of the purification was demonstrated by the higher expression of Wnt/β-catenin targets (*Axin2*, *Lef1* and *CyclinD1*) as well as *lacZ* from the *BAT*-*gal* reporter specifically in the β-cat^nc^ fraction relative to surrounding β-cat^m^ cells (Fig. 1d) [18]. In addition, expression of genes associated with embryonic and adult pituitary progenitors/stem cells, such as *Sox2* and *Nestin*, were also elevated in the β-cat^nc^ fraction [14, 20]. In contrast, expression of specific pituitary cell lineage commitment and terminal differentiation markers (*Pit1*, *Pomc* and *Gh*) was significantly lower in the β-cat^nc^ fraction. These data confirm our previous findings suggesting that the β-catenin-accumulating cells may correspond to undifferentiated pituitary progenitors/stem cells (PSC).

Next, we sought to explore this possibility more definitively using an in vitro approach. When cultured in stem-cell-promoting media, normal PSC form adherent colonies arising from single cells, and fitting an undifferentiated profile [20]. To explore if β-cat^nc^ clusters contain colony-forming progenitor/stem cells, flow-sorted β-cat^nc^ (BAT-gal^+ve^ fraction) and β-cat^m^ cells (BAT-gal^−ve^ fraction) were cultured for 1 week and assessed for colony formation. It was clear that total numbers of colony-forming cells were significantly increased in the fraction containing the β-cat^nc^ clusters (8.48-fold increase; Fig. 1e, f). However, only around 5% of the β-catenin-accumulating cells were capable of expanding to give rise to a colony under these conditions, suggesting that stemness is likely associated with a different factor, such as *Sox2* expression, rather than nucleocytoplasmic β-catenin accumulation.

**Fig. 2:**
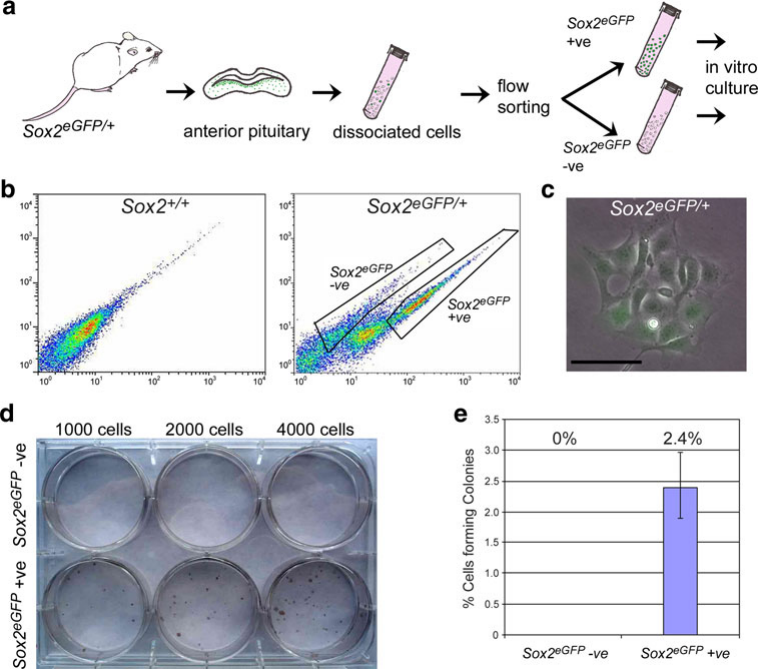
Pituitary progenitors/stem cells (PSCs) are contained in the SOX2-expressing population. **a** Scheme of cell purification strategy: *Sox2* ^*eGFP/*+^ postnatal pituitaries (P14) are dissociated into single cell suspensions and flow sorted to separate the eGFP^+ve^ and eGFP^−ve^ fractions. **b** Scatter plots showing the isolation of the two fractions. The gates used are indicated on the plot. **c** Clonal culture of cell preparations from unpurified *Sox2* ^*eGFP/*+^ pituitaries in stem cell-promoting media gives rise to eGFP^+ve^ colonies demonstrating the activation of the SOX2 promoter in PSCs. **d** Photograph of a tissue culture plate containing fixed and hematoxylin-stained colonies demonstrate the presence of colonies only in the flow sorted purified eGFP^+ve^ fraction. **e** Approximately 2.4% of the eGFP (*Sox2*)-expressing cells are able to form colonies. *Scale bar* 50 μm

To test this idea, we used a *Sox2*-*eGFP* mouse model, in which *Sox2*-expressing cells are marked by eGFP expression [12]. Flow-sorting purification of eGFP-expressing cells followed by culture in stem-cell-promoting media showed that only cells within the *Sox2*-expressing fraction were capable of generating colonies (Fig. 2a–d). Cells not expressing eGFP did not form colonies, strongly suggesting that PSCs are contained within the *Sox2*-expressing population (Fig. 2e).

**Fig. 3:**
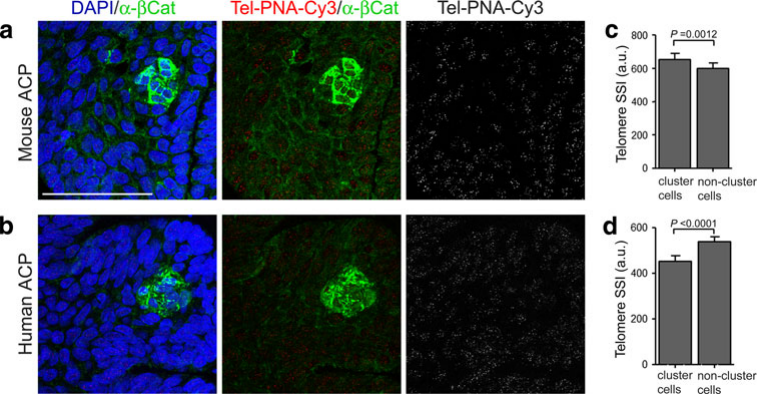
Analysis of telomere length in β-catenin accumulating clusters and surrounding cells in mouse and human ACP. (**a**, **b**) Telomere PNA FISH (Cy3 conjugate) and β-catenin immunofluorescence on mouse pre-tumoral lesions at 18.5 dpc (**a**) and human ACP samples (**b**) allows visualisation of telomeres specifically in β-catenin accumulating cluster cells or non-cluster surrounding cells. **c**, **d** Quantification of telomere length as sum spot intensity reveals significantly longer telomeres in β-catenin accumulating cluster cells in the mouse (**c**) whereas these are significantly shorter in the advanced human ACP sample (**d**). The pictures shown (**a**, **b**) are representative examples corresponding to single optical confocal sections of 0.001 μm thickness. Indicated statistics prepared using a Students *t*-test, *n* = 50 clusters for mouse, *n* = 25 clusters for human. *Scale bar* 50 μm

We have previously shown that in vivo β-cat^nc^ clusters in mouse and human ACP contain slow-dividing/quiescent cells, a feature often associated with stem cells [18]. Another feature linked to stemness is increased telomere length, as this gradually decreases with each cell cycle due to incomplete replication of telomeric DNA, but remains long in slow-dividing stem cells [15, 16]. Quantification of telomere length on histological sections of *Hesx1* ^*Cre/*+^; *Ctnnb1* ^*lox(ex3)/*+^ pituitaries at 18.5 dpc, showed that β-cat^nc^ cluster cells have significantly longer telomeres than the surrounding β-cat^m^ cells (Fig. 3a, c). However, a similar analysis on human ACP samples revealed longer telomeres in surrounding β-cat^m^ cells rather than β-cat^nc^ clusters (Fig. 3b, d). Together these data suggest that: (1) β-catenin-accumulating clusters in the murine ACP model exhibit a molecular profile and cellular behaviour characteristic of undifferentiated pituitary progenitors/stem cells; (2) β-catenin-accumulating clusters in human ACP, although slow-dividing and undifferentiated (i.e. lacking expression of terminal differentiation pituitary markers), do not express SOX2 or have longer telomeres and (3) mouse and human β-catenin-accumulating clusters may correspond to cells that are ontogenetically related but temporally distant; mouse cluster cells at 18.5 dpc relate to an early stage of tumour formation (pre-tumoral stage) whilst human cluster cells are present in clinically relevant and advanced tumours.

### Global gene expression analysis of β-cat^nc^ cluster cells from murine tumorigenic pituitaries reveals novel pathways involved in ACP

Having purified the β-cat^nc^ cluster cells from tumorigenic mouse pituitaries, we decided to carry out a global gene profiling study to reveal new genes/signalling pathways involved in human ACP.

Microarray analysis of flow-purified β-cat^nc^ cells in comparison with β-cat^m^ cells confirmed previously characterised gene expression changes [18]. As mentioned above, activation of the Wnt/β-catenin pathway targets *Axin2* and *Lef1* is restricted to cells within the β-cat^nc^ clusters in the *Hesx1* ^*Cre/*+^;*Ctnnb1* ^*lox(ex3)/*+*)*^ mouse model as well as in human ACP, assessed by in situ hybridisation. In the array data, levels were 16.77-fold higher for *Axin2*, 9.51-fold higher for *Lef1*, and *Sp5*, another universal Wnt/β-catenin target [56, 61] was found to be 12.69-fold higher in β-cat^nc^ clusters relative to the β-cat^m^ fraction (Supplementary Table 1). Likewise, expression of SOX2 is up-regulated in a proportion of β-cat^nc^ cells within the clusters, and in the array, *Sox2* levels were 1.62-fold higher in the cluster fraction. Conversely, the expression of terminal differentiation markers, such as *Pomc* (pro-opiomelanocortin-alpha) and *Gh* (growth hormone), both undetectable within β-cat^nc^ clusters by immunohistochemistry and with higher expression in surrounding β-cat^m^ cells by qRT-PCR, was specifically lower in the β-cat^nc^ cluster fraction by microarray comparison (9.01-fold lower for *Pomc* and 3.4-fold lower for *Gh*) (Supplementary Table 2). Changes in gene expression were further validated by quantitative real-time PCR (Supplementary Figure 1), demonstrating the efficiency of the cell purification and the robustness of the microarray data.

We interrogated the array data aiming to identify differentially expressed genes in the β-cat^nc^ cluster cells. This analysis revealed increased expression of signalling molecules involved in multiple cellular processes and physiological functions including normal development or stem cell maintenance and pathogenic conditions in humans such as tumorigenesis and cancer. These datasets have been deposited in ArrayExpress (Accession E-MEXP-3492) and are accessible to the scientific community. Here, we focus on a handful of genes and signalling pathways important for normal pituitary development or stem cell function, revealing an involvement of these in ACP.

The Hedgehog (HH) pathway participates in multiple developmental events and has been implicated in the maintenance of adult stem cell niches [7, 23, 39]. In addition, over-activation of the pathway has been identified in several human cancers as an important factor leading to tumour growth and metastasis [33, 40, 54]. When this pathway is down-regulated in the embryonic pituitary gland, progenitors of the Rathke’s pouch, the anterior pituitary primordium, fail to proliferate, resulting in a hypoplastic anterior pituitary but normal terminal differentiation of hormone-producing cells [59, 60]. *Shh* (sonic hedgehog) expression was found to be expressed 10.07-fold higher in β-cat^nc^ cluster cells (Supplementary Table 3).

The fibroblast growth factor (FGF) family and transforming growth factor (TGF) superfamily of secreted signalling molecules, including bone morphogenetic proteins (BMPs), are key regulators of several biological processes and together with their receptors, they affect the development of many human cancers [11, 21, 22, 62, 63]. In the pituitary gland, secreted FGFs and BMPs are required for normal specification and cell proliferation of pituitary progenitors during early pituitary development [10, 13, 36]. By microarray, members of the FGF family, such as *Fgf3*, *Fgf4* and *Fgf20* among others were expressed at higher levels in the β-cat^nc^ clusters relative to the surrounding β-cat^m^ cells (6.99-, 20.73- and 15.77-fold higher, respectively) (Supplementary Table 4). Likewise, expression of *Bmp2*, *Bmp4* and *Bmp7*, was also enhanced (3.72, 9.47, and 5.67-fold in the β-cat^nc^ clusters, respectively) (Suppl. Table 5). Other members of the TGFβ family were also expressed at much higher levels in these cluster cells: *Tgfa*, 5.25; *Tgfb1*, 5.75; *Tgfb2*, 3.54. Genes encoding subunits of activin dimers were also found to be up-regulated in β-cat^nc^ cells: *Inhba*, 7.27; *Inhbb*, 9.74 (Supplementary Table 6). Collectively, these molecular data strongly suggest that the β-cat^nc^ cells within the clusters play a non-cell autonomous role (i.e. autocrine and paracrine signalling), which may be relevant in the pathogenesis of ACP.

In addition, the pattern of expression of several chemokines not only confirmed a non-cell autonomous function, but also revealed a reciprocal signalling interaction between the β-cat^nc^ clusters and surrounding β-cat^m^ cells. Several members of the CXC and CC families of chemokines and their receptors were highly expressed in β-cat^nc^ cluster relative to the β-cat^m^ cells (Supplementary Table 7). Of relevance to this study, CXCR4, a seven-transmembrane span G-protein-coupled receptor expressed in multiple normal and cancer stem cells was expressed 2.63-fold higher in the cluster cells [42, 46]. Conversely, CXCL12 (also known as stromal-derived factor 1, SDF-1), which primarily binds to CXCR4 was expressed 4.92-fold higher in β-cat^m^ cells surrounding the β-cat^nc^ clusters. Likewise, some of the CC chemokine and CSF (colony-stimulating factor) receptors showed higher expression in the β-cat^nc^ cluster cells. In summary, the gene profiling analysis of β-cat^nc^ has revealed a specific molecular signature characterised by a great increase in the expression of multiple signalling molecules and, in addition, illustrates a complex reciprocal interaction between β-catenin-accumulating cluster cells and their surrounding cells in ACP tumorigenesis.

### Immunohistochemistry and in situ hybridisation studies demonstrate conserved novel pathways between mouse and human ACP

Several of the genes identified through array gene profiling of mouse ACP have not been previously implicated in the pathogenesis of human ACP. Therefore, we sought to validate the mouse array data described here with expression analysis for selected relevant genes on histological sections from both mouse and human ACP.

**Fig. 4:**
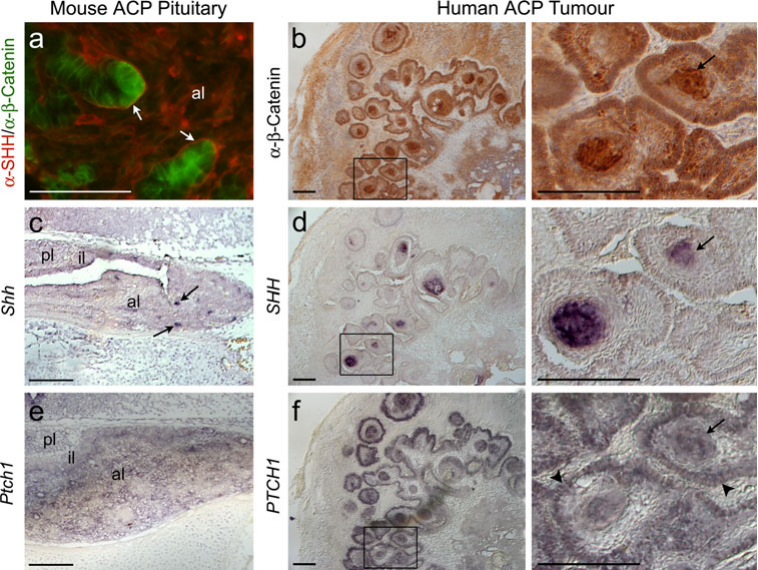
SHH signalling is active in mouse and human ACP. **a** Double immunofluorescence using antibodies against β-catenin (*green*) and SHH (*red*) showing the localisation of SHH in cells within the clusters of pituitaries from the ACP murine model at 18.5 dpc. Note the SHH protein on the cell membrane facing the stromal cells (*arrows*). **b** Immunohistochemistry reveals the presence of several β-catenin-accumulating cell clusters on a human ACP sample (*arrows*). **c**, **d** In situ hybridisation on the ACP mouse model (**c**) and human ACP (**d**) with *Shh* antisense riboprobes showing the up-regulation of *Shh/SHH* in some of the β-catenin-accumulating cell clusters (*arrows*). **e**, **f** In situ hybridisation on the ACP mouse model (**e**) and human ACP (**f**) with *Ptch1* antisense riboprobes. *Ptch1*, a target of SHH signalling, is expressed throughout the pituitary of the mouse model and in both the palisading cells (*arrowheads*) and the β-catenin-accumulating cell clusters (*arrows*) in the human ACP sample. *Scale bars* **a**, **b**, **d**, **f**, 50 μm; **c**, **e** 150 μm

Hedgehog-secreted signals (e.g. SHH) bind to the receptor Patched 1 (*Ptch1*), leading to the de-repression of the transducer Smoothened (*Smo*). Activated SMO initiates the intracellular signalling cascade and the nuclear translocation of GLI, ultimately causing the transcriptional activation of target genes, including *Gli1* and *Ptch1* [30]. In *Hesx1* ^*Cre/*+^;*Ctnnb1* ^*lox(ex3)/*+^ tumorigenic pituitaries, the *Shh* expression pattern was very similar to that observed for β-catenin, i.e. in distinct foci within the anterior and intermediate lobes, suggesting the likely co-expression of these genes in the β-cat^nc^ cell clusters (Fig. 4c). Immunohistochemistry with SHH antibodies revealed specific staining in the cytoplasmic membrane of the vast majority of pituitary cells, as expected for a secreted molecule of lipophilic nature. Within the clusters, SHH protein was detected only on the basal surface of the cells, i.e. the surface limiting the clusters and surrounding cells, indicating polarized secretion outside the clusters (Fig. 4a). To identify the responding cells where the HH pathway was activated, we analysed expression of *Ptch1*, the SHH receptor and a target of the pathway. In *Hesx1* ^*Cre/*+^; *Ctnnb1* ^*lox(ex3)/*+^ pituitaries, strong signal was broadly detected throughout the anterior lobe with some regions of stronger expression (Fig. 4e). Together, these data indicate autocrine as well as paracrine SHH signalling activation in the β-cat^nc^ cluster and β-cat^m^ cells, respectively.

Next, we investigated whether the expression pattern of relevant HH signalling pathway components may be conserved in human ACP. A tentative role for this pathway in human ACP was proposed in a familial case of Gorlin syndrome showing the typical phenotype (basal cell carcinoma and craniofacial and bone malformations) in association with ACP and harbouring a mutation in *PTCH1*, but neither *SHH* nor *PTCH1* expression could be identified in this study [45]. In situ hybridisation on human ACP sections (*n* = 5) showed a pattern of *SHH* expression characterised by the presence of cell clusters analogous to the typical β-cat^nc^ cell clusters observed in these human tumours (Fig. 4d). Analysis on consecutive sections with SHH riboprobes and α-β-Catenin antibody revealed the co-expression of these two genes, as observed in the mouse model (Fig. 4b, d). Finally, expression of *PTCH1* was identified in cluster cells as well as in the palisading cells surrounding these typical lesions (Fig. 4f). These findings not only further validate our mouse model for the study of human ACP, but also demonstrate that the HH pathway is also active in human ACP in both the β-catenin-accumulating cell clusters, which secrete the SHH ligand, as well as in some surrounding β-cat^m^ cells.

**Fig. 5:**
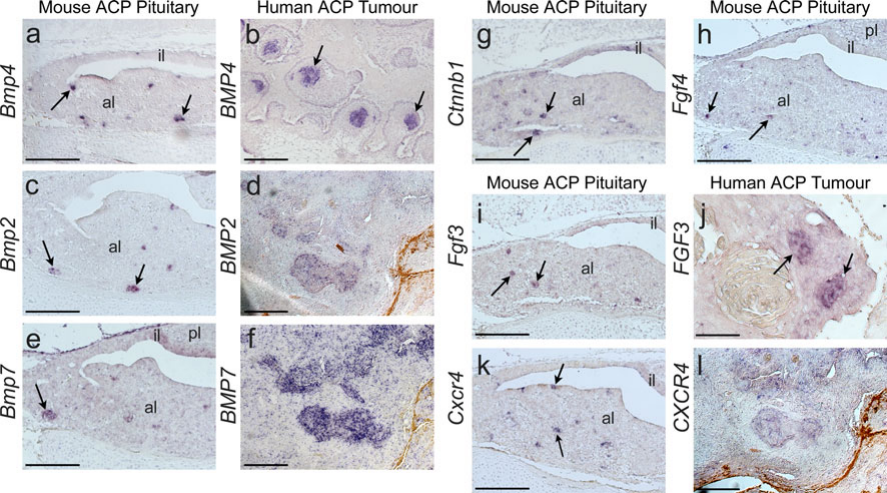
Expression analysis by in situ hybridisation of mouse and human ACP. Mouse pituitaries at 18.5 dpc. **a**–**f** Expression of *Bmp/BMP* members 4, 2 and 7 is up-regulated in pre-tumoral mouse pituitaries at 18.5 dpc and human ACP (*arrows*). In the ACP mouse model pituitary, expression is mainly restricted to cell clusters. In human ACP, expression is up-regulated in cell clusters, but other tumour cells express *BMP2* and *BMP7*. **g**, **h** *Ctnnb1* and *Fgf4* expression in the ACP mouse model pre-tumoral pituitary is up-regulated in cluster cells (*arrows*). **i**, **j** *Fgf3/FGF3* expression is also up-regulated in cluster cells in both mouse and human ACP (*arrows*). **k**, **l** *Cxcr4* expression is detected in clusters in the ACP mouse model pituitary (*arrows*), but it is more widely expressed in human ACP. *Scale bars* **a**, **c**, **e**, **g**, **h**, **i**, **k**, 150 μm; **b**, **d**, **f**, **j**, **l** 50 μm

Data from the array study showed elevated expression of *Bmp2*, *Bmp4* and *Bmp7* in the β-cat^nc^ clusters compared with non-cluster β-cat^m^ cells. This was confirmed by in situ hybridization analysis on *Hesx1* ^*Cre/*+^;*Ctnnb1* ^*lox(ex3)/*+^ tumorigenic pituitaries, which revealed a pattern of expression of these genes in restricted focal points reminiscent of the β-catenin accumulating clusters (Fig. 5a, c, e). In human ACP, *BMP4* has previously been shown to have increased expression within the β-cat^nc^ clusters [29]. We confirmed this finding and revealed a comparable expression pattern for *BMP2* and *BMP7* in human ACP samples (Fig. 5b, d, f). Expression of *Fgf3* and *Fgf4* was observed in the mouse β-cat^nc^ clusters, and we were able to confirm *FGF3* expression also in the human clusters (Fig. 5g–j).

Finally, we analysed the expression pattern of *Cxcr4* because of its known expression in numerous normal as well as cancer stem cell populations and its proposed role in cell migration and metastatic infiltration [6, 42, 46]. An activated CXCL12/CXCR4 axis has also been associated with proliferation of pituitary adenomas [4, 5]. In situ hybridization showed strong *Cxcr4* expression in restricted foci resembling the β-cat^nc^ cell clusters in mouse tumorigenic pituitaries (Fig. 5k). In human ACP samples, CXCR4 expression was not very abundant and broadly expressed throughout the tumour with weak up-regulation in some clusters (Fig. 5l). Overall, this expression analysis confirmed the array data and supports the concept that β-catenin-accumulating cells in mouse and human ACP function as signalling centres from where multiple secreted signals emanate to act on themselves and neighbouring cells.

## Discussion

In this paper, we have utilised a recently generated mouse model for ACP and carried out an unbiased molecular screen demonstrating the de-regulation of numerous genes and signalling pathways with tumorigenic potential in both mouse and human ACP. We provide molecular and expression data indicating that β-cat^nc^ cluster cells act as a source of mitogenic and pro-survival signals for themselves and surrounding β-cat^m^ tumour cells. Our global gene profiling analysis has revealed that members of the SHH, FGF and BMP family of morphogens, which are critical during normal pituitary development [23, 30, 39], show higher expression levels in the β-cat^nc^ cluster cells in both mouse and human ACP. As well as providing novel insights into the pathogenesis of human ACP, this research has identified potential therapeutic targets for these tumours.

SHH is required during the normal development of several organs and in adulthood this pathway plays an important role in the maintenance of stem-cell niches [1, 30, 53]. Over-active HH signalling occurs in numerous human cancers and can be caused by either a mutation-driven (ligand-independent) mechanism or ligand-dependent signalling (i.e. autocrine/paracrine signalling). Inactivating mutations in PTCH1 or more rarely, activating mutations in SMO have been identified in most sporadic medulloblastomas [19, 48, 64]. Loss-of-function mutations in PTCH1 underlie the molecular cause of Gorlin syndrome (also known as nevoid basal cell carcinoma syndrome), a rare condition characterised by an increased risk of developing various tumours, commonly medulloblastoma, rhabdomyosarcoma and basal cell carcinoma [58]. In contrast, many epithelial cancers, including small-cell lung cancer, pancreatic, prostate and gastrointestinal malignancies, also exhibit over-active HH pathway, but this is caused by increased expression of SHH ligand without known mutations in pathway components. In these tumours, SHH is released to the stroma (paracrine action) where it promotes tumour growth, infiltration and angiogenesis. In addition, activation of the SHH pathway in stromal cells is proposed to feedback signals to induce a more suitable environment for the SHH-expressing cells [58]. SHH autocrine signalling within the epithelial cells also appears to be important for cell renewal of cancer stem cells in breast cancer, multiple myeloma and chronic myelogenous leukaemia stem cells [64]. Our expression data strongly support a key role for HH signalling in the pathogenesis of mouse and human ACP, whereby SHH expression in the β-cat^nc^ cell clusters activates the pathway in both β-cat^nc^ (autocrine action) and β-cat^m^ cells (paracrine signalling). Whether over-activating mutations in the HH pathway components may underlie ACP tumorigenesis requires further research.

Over-activating mutations in FGF receptors have been identified in several human cancers, including breast, bladder, prostate, endometrial and lung cancers as well as haematological malignancies [62]. Expression levels of the FGF receptors 1–4 remained essentially unchanged between β-cat^nc^ clusters and surrounding β-cat^m^ cells. We noticed, however, that expression of *Fgfrl1* (FGF receptor like-1), a recently identified receptor lacking the critical intracellular domain responsible for signal transduction and thought to act as a decoy receptor able to inhibit FGF signalling in the expressing cells [55], was expressed 8.99-fold higher in the cluster cells. Although bestowed with several functional activities, FGFs are potent mitogenic signals in a variety of cell contexts, including the pituitary gland. It is tempting to speculate that β-cat^nc^ cell clusters may act as a source of FGFs inducing surrounding β-cat^m^ cells to actively divide, while protecting themselves by expressing higher levels of *Fgfrl1*. This could explain the paradoxical observation that cluster cells in both mouse and human ACP remain quiescent (e.g. Ki67 negative), but cells in the immediate vicinity are mitotically active [18]. Related to this notion, β-cat^nc^ cells express high levels of anti-apoptotic proteins of the Bcl2/Bcl-xL family.

An interesting concept that can be inferred from our data relates to the origin of the β-catenin accumulating (β-cat^nc^) cluster cells. In mouse, these cells have an embryonic origin in Rathke’s pouch undifferentiated precursors (i.e. HESX1 and SOX2 expressing cells) [18]. However, SOX2 is not uniformly expressed in all clusters or every cell within the clusters in the mouse pre-tumoral pituitary at late gestation or early postnatal life (Fig. 1b) [18]. Subsequently, in advanced mouse tumours, SOX2 expression is rarely observed, although at this stage, β-cat^nc^ clusters are not identifiable and most of the tumour cells exhibit accumulation of β-catenin in the nucleus, cytoplasm or both [18]. Similarly, although SOX2 positive cells have been identified in human ACP [17] (our unpublished observations), they are not present in the β-cat^nc^ cluster cells. This suggests that β-cat^nc^ cluster cells in human ACP and β-catenin-accumulating cells in advanced mouse ACP may derive from undifferentiated precursors/stem cells present in the embryonic or postnatal pituitary, but have lost cell stemness and down-regulated SOX2 expression.

Our data on telomere length on mouse and human β-cat^nc^ cluster cells are compatible with the idea that this similar cellular component of the tumours may correspond to different temporal stages of ACP development. Mouse β-cat^nc^ cluster cells in the pre-tumoral pituitaries exhibit longer telomeres than surrounding β-cat^m^ cells, suggesting the presence of stem cells in these structures. In contrast, β-cat^nc^ cluster cells in fully established human ACP have shorter telomeres than the rest of the non-cluster β-cat^m^ cells, suggesting that they do not contain stem cells. This could indicate the cellular ontogenesis of the β-cat^nc^ clusters in ACP, whereby SOX2^+ve^ stem cells with long telomeres are initially present but differentiate at later stages of tumour development losing SOX2 expression and shortening their telomeres. Unfortunately, species differences in ACP prevents us from testing this idea experimentally as fully established tumours in our mouse model do not contain β-cat^nc^ clusters and early stages of human ACP are not available.

Regardless, it is tempting to speculate that human ACP may be a tumour of stem cell origin, in which pituitary progenitors/stem cells played a role solely at an early stage of tumorigenesis. Advanced and clinically relevant human ACP would be devoid of such cells. This is different from the general dogma for cancer stem cells, where they self-renew and give rise to progeny that populate the tumour bulk. Instead, the contribution of the stem cell could be to initiate a cascade of signalling events leading to the perpetuation of a pathogenic unit (β-cat^nc^ cluster cells and microenvironment), without further need for the original stem cells. Therefore, the tumour-initiating mutation may occur in a progenitor/stem cell but the propagation of the tumour may require a different cell type. This is in agreement with findings in other tumours such as glioma and medulloblastoma [37, 50]. The data presented here strongly suggest an important autocrine/paracrine function of the cluster cells as signalling centres within the tumour and the interplay with the stromal cells, which is maintained after loss of SOX2 expression.

Current treatments for human ACP are far from ideal and associated with high morbidity and significant mortality [31, 43]. Our data highlight several genes and pathways likely to play essential roles in the pathogenesis of human ACP, as they do in several other human cancers. For some of these pathways, specific small-molecule inhibitors have been designed and their efficacy is currently being tested in a variety of clinical trials [35, 57]. The research presented here is expected to promote the development of chemical-based therapies leading to more efficient and safer treatments for these childhood tumours.

## Electronic supplementary material

Below is the link to the electronic supplementary material.

Supplementary material 1 (DOCX 25 kb)

Supplementary material 2 (DOCX 31 kb)

Supplementary material 3 (EPS 20013 kb)
