# Supplementary material for: Identification of novel pathways involved in the pathogenesis of human adamantinomatous craniopharyngioma
Source: Acta Neuropathol. 2012 Feb 18;124(2):259–71. doi: 10.1007/s00401-012-0957-9 (PMC3400760; doi:10.1007/s00401-012-0957-9)
Supplement: Supplementary file 1 — Supplementary material 1 (DOCX 25 kb) [file 401_2012_957_MOESM1_ESM.docx]

**Table 1. Fold changes in components of the Wnt signalling pathway.**

| **Fold Change Enrichment in Clusters** | **Gene Symbol** | **Affymetrix Probe Set** |
| --- | --- | --- |
| 16.771511 | *Axin2* | 1421341_at |
| 12.694851 | *Sp5* | 1422914_at |
| 9.508693 | *Lef1* | 1454734_at |
| 1.79152 | *Ccnd1* | 1448698_at |
|  | | |
| 4.9141965 | *Wnt6* | 1419708_at |
| 2.6935523 | *Wnt5a* | 1448818_at |
| 1.6087853 | *Wnt16* | 1422941_at |
| 1.5826728 | *Wnt4* | 1450782_at |
| 1.5687882 | *Wnt9b* | 1443197_at |
| 1.4131924 | *Wnt2* | 1449425_at |
| 1.3493328 | *Wnt11* | 1450772_at |
| 1.2245699 | *Wnt8b* | 1421439_at |
| 1.1444898 | *Wnt7a* | 1423367_at |
| 1.1313456 | *Wnt8a* | 1422228_at |
| 1 | *Wnt7b* | 1420891_at |
| -1.0491053 | *Wnt9a* | 1425889_at |
| -1.0973257 | *Wnt2b* | 1421465_at |
| -1.3130513 | *Wnt3a* | 1422093_at |

Positive fold changes denote higher expression in β-cat^nc^ clusters. Negative values indicate higher expression in β-cat^m^ cells.

**Table 2. Fold changes in pituitary markers.**

| **Fold Change Enrichment in Clusters** | **Gene Symbol** | **Affymetrix Probe Set** |
| --- | --- | --- |
| 1.6200087 | *Sox2* | 1416967_at |
| -2.0506825 | *Sox9* | 1451538_at |
| -3.400096 | *Gh* | 1460613_x_at |
| -3.9543037 | *Pou1f1* | 1422220_at |
| -4.8568234 | *Cga* | 1418549_at |
| -9.078063 | *Pomc* | 1433800_a_at |
| -33.148746 | *Tshb* | 1450371_at |

Positive fold changes denote higher expression in β-cat^nc^ clusters. Negative values indicate higher expression in β-cat^m^ cells.

**Table 3. Fold changes in components of the hedgehog signalling pathway**.

| **Fold Change Enrichment in Clusters** | **Gene Symbol** | **Affymetrix Probe Set** |
| --- | --- | --- |
| 10.069663 | *Shh* | 1436869_at |
| 1.3864627 | *Gli3* | 1455154_at |
| 1.2886769 | *Ptch1* | 1428853_at |
| -1.0722258 | *Gli2* | 1459211_at |
| -1.0809623 | *Gli1* | 1449058_at |
| -1.2428762 | *Bcl2* | 1422938_at |
| -1.7886883 | *Smo* | 1427048_at |

Positive fold changes denote higher expression in β-cat^nc^ clusters. Negative values indicate higher expression in β-cat^m^ cells.

**Table 4. Fold changes in components of the FGF family.**

| **Fold Change Enrichment in Clusters** | **Gene Symbol** | **Affymetrix Probe Set** |
| --- | --- | --- |
| 20.7271 | *Fgf4* | 1449729_at |
| 15.768551 | *Fgf20* | 1421677_at |
| 6.9983816 | *Fgf3* | 1441350_at |
| 4.711376 | *Fgf15* | 1418376_at |
| 2.6798372 | *Fgf17* | 1456239_at |
| 2.335208 | *Fgf9* | 1420795_at |
| 2.049318 | *Fgf18* | 1449545_at |
| 1.4106169 | *Fgf6* | 1427582_at |
| 1.1066791 | *Fgf13* | 1418498_at |
| 1.0190424 | *Fgf22* | 1450205_at |
| -1.028142 | *Fgf11* | 1421793_at |
| -1.0330502 | *Fgf1* | 1423136_at |
| -1.0332971 | *Fgf21* | 1422916_at |
| -1.2175977 | *Fgf16* | 1420806_at |
| -1.3176153 | *Fgf5* | 1438883_at |
| -1.5680567 | *Fgf7* | 1422243_at |
| -1.5797077 | *Fgf10* | 1420690_at |
| -1.6213022 | *Fgf14* | 1435747_at |
| -1.8110296 | *Fgf23* | 1422176_at |

Positive fold changes denote higher expression in β-cat^nc^ clusters. Negative values indicate higher expression in β-cat^m^ cells.

**Table 5. Fold changes in components of the BMP family.**

| **Fold Change Enrichment in Clusters** | **Gene Symbol** | **Affymetrix Probe Set** |
| --- | --- | --- |
| 9.4739065 | *Bmp4* | 1422912_at |
| 5.6704154 | *Bmp7* | 1418910_at |
| 3.717977 | *Bmp2* | 1423635_at |
| 1.9359876 | *Bmp8b* | 1440706_at |
| 1.7861356 | *Bmp5* | 1455851_at |
| 1.5066067 | *Bmpr2* | 1434310_at |
| 1.401446 | *Bmp10* | 1421763_at |
| 1.245159 | *Bmpr1b* | 1422872_at |
| 1.0601007 | *Bmp15* | 1420006_at |
| 1 | *Bmp8a* | 1449873_at |
| -1.037945 | *Bmp5* | 1421282_at |
| -1.039795 | *Bmp6* | 1450759_at |
| -1.2558248 | *Bmpr1a* | 1425491_at |
| -1.3214427 | *Bmp1* | 1426238_at |

Positive fold changes denote higher expression in β-cat^nc^ clusters. Negative values indicate higher expression in β-cat^m^ cells.

**Table 6. Fold changes in components of the TGFβ family.**

| **Fold Change Enrichment in Clusters** | **Gene Symbol** | **Affymetrix Probe Set** |
| --- | --- | --- |
| 5.747224 | *Tgfb1* | 1420653_at |
| 5.2426987 | *Tgfa* | 1421943_at |
| 3.5432453 | *Tgfb2* | 1450923_at |
| -1.5727837 | *Tgfb3* | 1417455_at |
| 9.740904 | *Inhbb* | 1426858_at |
| 7.2711034 | *Inhba* | 1422053_at |

Positive fold changes denote higher expression in β-cat^nc^ clusters. Negative values indicate higher expression in β-cat^m^ cells.

**Table 7. Fold changes in chemokines and their receptors.**

| **Fold Change Enrichment in Clusters** | **Gene Symbol** | **Affymetrix Probe Set** |
| --- | --- | --- |
| 2.6252391 | *Cxcr4* | 1448710_at |
| 1.2083099 | *Cxcr6* | 1422812_at |
| -1.2477317 | *Cxcr5* | 1422003_at |
| -1.5073917 | *Cxcr3* | 1449925_at |
| 30.06548 | *Cxcl2* | 1449984_at |
| 29.647043 | *Cxcl3* | 1438148_at |
| 7.9567375 | *Cxcl10* | 1418930_at |
| 4.0969024 | *Cxcl15* | 1456428_at |
| 2.6390684 | *Cxcl14* | 1418457_at |
| 2.154167 | *Cxcl1* | 1419209_at |
| 1.8197103 | *Cxcl5* | 1419728_at |
| 1.4014463 | *Cxcl9* | 1456907_at |
| 1.1240754 | *Cxcl16* | 1418718_at |
| 1.0937543 | *Cxcl11* | 1419697_at |
| -1.2101607 | *Cxcl13* | 1417851_at |
| -1.3426214 | *Cxcl17* | 1451610_at |
| -4.91697 | *Cxcl12* | 1417574_at |

Positive fold changes denote higher expression in β-cat^nc^ clusters. Negative values indicate higher expression in β-cat^m^ cells.
