# Supplementary material for: Identification of novel pathways involved in the pathogenesis of human adamantinomatous craniopharyngioma
Source: Acta Neuropathol. 2012 Feb 18;124(2):259–71. doi: 10.1007/s00401-012-0957-9 (PMC3400760; doi:10.1007/s00401-012-0957-9)
Supplement: Supplementary file 2 — Supplementary material 2 (DOCX 31 kb) [file 401_2012_957_MOESM2_ESM.docx]

**Supplementary Fig. 1** qRT-PCR analysis of β-catenin-accumulating mouse clusters versus surrounding cells. Positive values denote higher expression in the former and negative values indicate higher expression in the latter.
